# The Effect of Elevated Triglycerides on the Onset and Progression of Coronary Artery Disease: A Retrospective Chart Review

**DOI:** 10.1155/2015/292935

**Published:** 2015-11-04

**Authors:** Deepu Daniel, Patrick Hardigan, Asif Jawaid, Rohit Bhandari, Mithun Daniel

**Affiliations:** ^1^Broward Health Medical Center, 1600 South Andrews Avenue, Fort Lauderdale, FL 33316, USA; ^2^Health Professions Division's Statistical Consulting Center, Nova Southeastern University, 3200 South University Drive, Davie, FL 33328, USA; ^3^United Memorial Medical Center, 127 North Street, Batavia, NY 14020, USA

## Abstract

*Background*. The American College of Cardiology and American Heart Association did not indicate a correlation between treating hypertriglyceridemia and reducing cardiovascular events. *Objective*. This study investigated whether patients with hypertriglyceridemia were more prone to worse outcomes during cardiac catheterization.* Methods*. Data collected over a one-year period analyzed lipid panels obtained at the time of cardiac catheterization. Triglyceride levels were categorized into three groups: <150 mg/dL, 150 mg/dL–300 mg/dL, and >300 mg/dL. Controlled variables included age, gender, the presence of hypertension, diabetes, hyperlipidemia, and history of coronary artery disease. *Results*. Subjects with a triglyceride level <150 mg/dL have a 54% likelihood of being treated medically compared to 38% and 41% in the 150 mg/dL–300 mg/dL and >300 mg/dL groups, respectively (*p* < 0.01). Subjects with a triglyceride level >300 mg/dL have a 20% percent chance of being treated with a coronary artery bypass graft compared to 12% and 15% in the <150 mg/dL and 150 mg/dL–300 mg/dL groups, respectively (*p* < 0.01). Subjects with a triglyceride level between 150 and 300 mg/dL have a 44% percent of being treated with a percutaneous coronary intervention compared to 34% and 43% in the <150 mg/dL and >300 mg/dL groups, respectively (*p* < 0.01). *Conclusion*. Hypertriglyceridemia was associated with worse outcomes in percutaneous coronary intervention or surgery.

## 1. Introduction

The most recent ACC/AHA guidelines did not make any specific recommendations on the treatment of elevated triglycerides in regard to decreasing risk of heart disease [1]. It suggested that triglycerides of greater than 500 mg/dL should prompt investigation of secondary causes of hyperlipidemia, but the guidelines did not show any additional reduction in cardiovascular risk with the treatment of these elevated levels. This differs from the ESC/ESA guidelines on the management of dyslipidemia [2]. These societies, along with the Joint European guidelines, do in fact identify elevated triglycerides as an important cardiovascular disease risk factor [3].

This team believes that elevated triglycerides should be given more careful consideration for earlier, aggressive treatment in order to prevent coronary artery disease. The study analyzed all the cardiac stress tests done in the hospital over a one-year period along with the patients' corresponding lipid levels. The purpose of the study was to determine if patients with higher triglyceride level were more prone to have worse results with cardiac catheterization. The team controlled for 6 different variables: age, gender, the presence of diabetes, hypertension, LDL levels, and history of coronary artery disease. The data was collected over a one-year period.

## 2. Materials and Methods

Descriptive statistics were calculated for all study variables. This includes the mean and standard deviation for continuous measures and frequencies for categorical outcomes. A probability value 0.05 was considered statistically significant, and all tests were two-tailed. The statistical packages STATA V14.0 and R 3.1.2 were used in all statistical analyses. Pairwise comparisons were employed with a Bonferonni adjustment.

The primary outcome variable was type of treatment: (a) medical management, (b) coronary artery bypass grafting or CABG, and (c) percutaneous coronary intervention or PCA/PCI. The specific variable of interest was triglyceride levels which was categorized into the following levels: (a) <150 mg/dL, (b) 150–300 mg/dL, and (c) >300 mg/dL. The model covariates included a subject's age, gender, low-density lipoprotein cholesterol levels, and diabetic status (yes or no), hypertensive status (yes or no), and coronary artery disease status (yes or no). To examine the relationship between triglyceride levels and type of treatment, a multinomial logistic regression model was created.

## 3. Results

Data from one thousand, four hundred and fifty-one subjects were used in the analysis. Simple bivariate statistical analysis reveals significant differences between the groups across all six covariates. For example, the medical intervention groups are younger, more likely to be female, and less likely to be diabetic or possess coronary arty disease (Table 1). As such, all covariates were used in the statistical model.

Results from the multinomial model for the covariates indicate that men are 3.23 [95% CI (2.19–4.76), *p* < 0.000] times more likely to be given a CABG and 2.19 [95% CI (1.37–2.29), *p* < 0.000] times more likely to be given PCA/PCI than women. Additionally, the model for the medical covariates indicates with the following:

(i) Diabetics are 1.97 [95% CI (1.38–2.80), *p* < 0.000] times more likely to be given a CABG and 1.32 [95% CI (1.02–1.71), *p* = 0.034] times more likely to be given PCA/PCI than nondiabetics.
(ii) Subjects without hypertension are 1.33 [95% CI (1.02–1.75), *p* = 0.042] times more likely to be given PCA/PCI than subjects with hypertension.
(iii) Subjects with cardiovascular disease are 2.36 [95% CI (1.83–3.05), *p* < 0.000] times more likely to be given PCA/PCI than subjects without cardiovascular disease.
(iv) For every one unit increase in low-density lipoprotein cholesterol level, the relative risk of a subject receiving a CABG increases with 1.01 [95% CI (1.00–1.02), *p* < 0.000] and a subject receiving a PCA/PCI increases with 1.01 [95% CI (1.00–1.02), *p* < 0.000].

The multinomial logistic model indicates a significant difference between the groups based on triglyceride levels while controlling for the study covariates (Table 2). Additionally, a relationship is revealed between triglycerides and type of treatment. Subjects with triglyceride levels less than 150 mg/dL are most likely to be treated medically, subjects with a triglyceride level between 150 and 300 mg/dL are most likely to be given a PCA/PCI, and subjects with a triglyceride level greater than 300 mg/dL are most likely to be given a CABG (Table 3). To better communicate this model we break down the results by the outcome variable.

**Figure 1:**
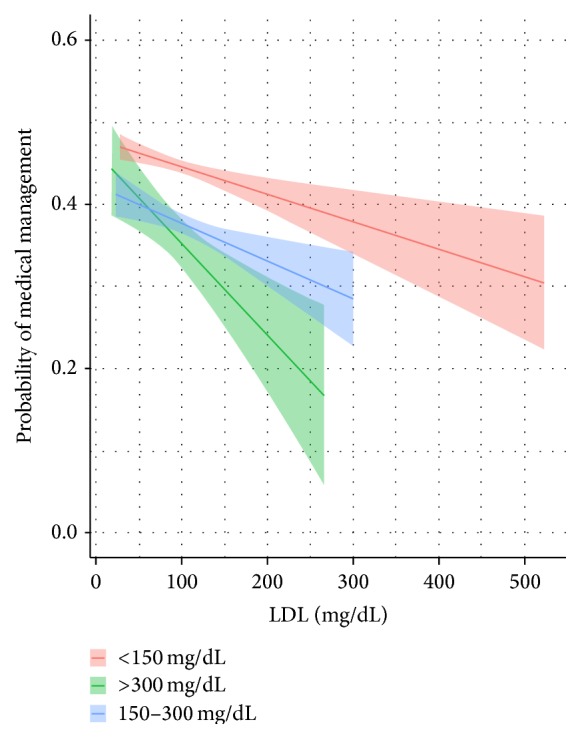
Predicted probability with 95% confidence levels of a patient treated medically by triglyceride level.

*Medical Management*. Controlling for the six covariates, subjects with a triglyceride level less than 150 mg/dL have a 54% percent of being treated medically. This compares to the 150–300 mg/dL and >300 mg/dL groups who possess 38 and 41 percent chance, respectively. Pairwise comparisons indicate the following significant differences (*p* < 0.01)—see Figure 1:

1. 150 mg/dL versus 150–300 mg/dL: 12.8% [95% CI: 10.6% to 14.9%];
2. 150 mg/dL versus >300 mg/dL: 16.1% [95% CI: 11.2% to 21.0%].

**Figure 2:**
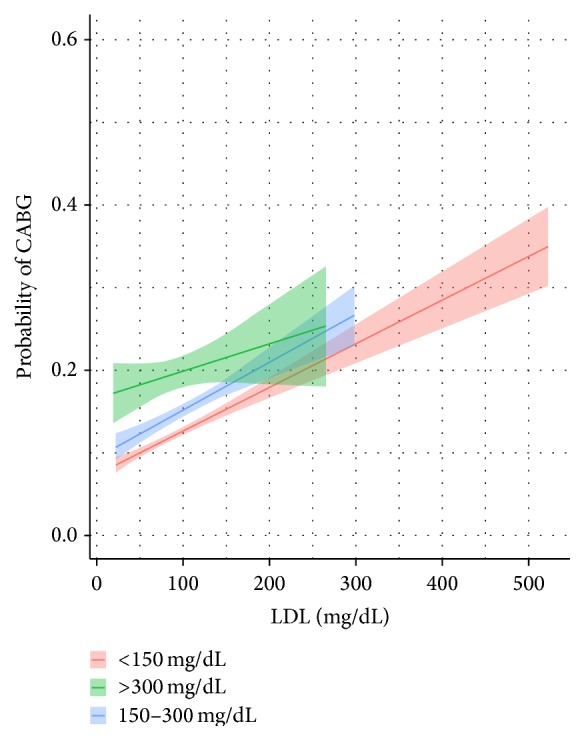
Predicted probability with 95% confidence levels of a patient treated with a CABG by triglyceride level.

*CABG*. Controlling for the six covariates, subjects with a triglyceride level greater than 300 mg/dL have a 20% percent of being treated with a CABG. This compares to the <150 mg/dL and 150 mg/dL to 300 mg/dL groups who possess 12 and 15 percent chance, respectively. Pairwise comparisons indicate the following significant differences (*p* < 0.01)—see Figure 2:

1. >300 mg/dL versus <150 mg/dL: 7.1% [95% CI: 1.9% to 4.0%];
2. >300 mg/dL versus 150–300 mg/dL: 4.1% [95% CI: 1.7% to 6.7%].

**Figure 3:**
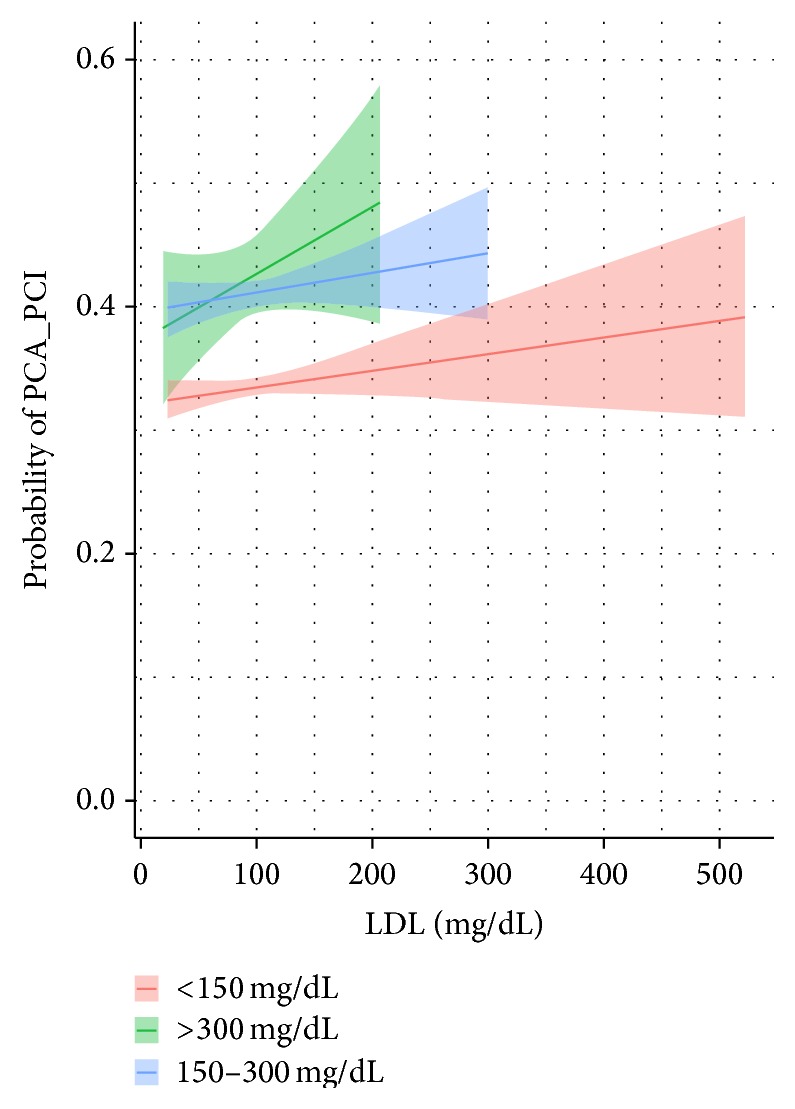
Predicted probability with 95% confidence levels of a patient treated with a PCA/PCI by triglyceride level.

*PCA/PCI*. Controlling for the six covariates, subjects with a triglyceride level between 150 and 300 mg/dL have a 44% percent of being treated with a PCA_PCI. This compares to the <150 mg/dL and >300 mg/dL groups who possess 34 and 43 percent chance, respectively. Pairwise comparisons indicate the following significant differences (*p* < 0.01)—see Figure 3:

1. 150–300 mg/dL versus <150 mg/dL: 9.8% [95% CI: 8.1% to 11.4%].

## 4. Discussion

The 2013 ACC/AHA guidelines on the management of hyperlipidemia introduced a new method to asses and treat patients with elevated cholesterol levels. It first outlined four major categories of patients that would ultimately benefit the most from statin therapy. These four groups included those with clinical atherosclerotic cardiovascular disease [4, 5], diabetics [6, 7], and low-density lipoprotein (LDL) levels greater than 190 mg/dL [8, 9] and those with a ten-year atherosclerotic cardiovascular disease (ASCVD) risk score of greater than 7.5% [10, 11]. There is only a brief mention of evaluating and treating triglycerides if in excess of 500 mg/dL [12]. However, the panel was unable to provide concrete evidence that therapy with a nonstatin drug led to any further reduction in ASCVD risk [13]. These guidelines did not discuss in detail the role of triglyceride reduction in preventing cardiovascular morbidity and mortality. There are, however, studies which demonstrate that hypertriglyceridemia is a cardiovascular disease risk factor [14, 15].

This study investigated the hypothesis that patients with elevated triglycerides would be at higher risk of worse outcomes during cardiac catheterizations. The three results possible at cardiac catheterization were the need for angioplasty and/or cardiac stent placement, the need for coronary artery bypass graft, and finally medical therapy alone. Patients' triglyceride levels were categorized into less than 150 mg/dL, between 150 mg/dL and 300 mg/dL, and those greater than 300 mg/dL.

Over 1400 hundred patients (*n* = 1451) were included in this retrospective chart review, taken from a large tertiary county hospital over a one-year period (January 1st to December 31st 2011). Patients' preoperative lipid panels before cardiac catheterization were examined for triglyceride levels. Variables included were age, LDL, history of diabetes, hypertension, and previous history of coronary artery disease.

The results of the study showed that patients with elevated triglyceride levels, controlling for the above-mentioned variables, were more likely to undergo either stent placement or coronary artery bypass graft versus just medical therapy. Patients with triglyceride levels less than 150 mg/dL had a 54% chance of being treated medically compared to 38% and 41% in the 150–300 mg/dL and greater than 300 mg/dL groups, respectively (*p* < 0.01). The study also found that patients with triglyceride levels greater than 300 mg/dL had a 20% chance of undergoing CABG versus 12% in the <150 mg/dL group and 15% in the 150–300 mg/dL group (*p* < 0.01). It also indicated that patients with triglycerides 150–300 mg/dL had a 44% chance of receiving angioplasty and/or cardiac stent, while patients with levels <150 mg/dL had a 34% chance and those with levels >300 mg/dL had a 43% chance of PCA/PCI (*p* < 0.01). All of the aforementioned results were obtained controlling for the age, history of diabetes, hypertension, LDL, and previous history of coronary artery disease.

The research group believes that these results show the importance of elevated triglycerides toward the development and/or progression of coronary artery disease. This study shows that patients with elevated triglyceride levels were more likely to undergo cardiac stent placement and CABG compared to those with normal triglycerides, who were more likely to be managed through medical therapy instead. For the primary care setting, this study shows that triglycerides are important to monitor and treat in order to decrease cardiac morbidity and mortality. Considering the annual cost of hospitalization and medical care secondary to coronary artery disease and its complications and the relatively inexpensiveness and availability of a fasting lipid panel, triglyceride monitoring makes feasible sense.

## 5. Conclusion

This research study shows the importance of elevated triglycerides toward the onset and/or progression of coronary artery disease as evaluated during cardiac catheterizations. Results indicate that patients with elevated triglyceride levels had worse outcomes, more prone to stent placement and CABG. These results were statistically significant, with a *p* value <0.01. The message for primary care physicians is that patients with elevated triglycerides should be more aggressively monitored and treated in order to prevent coronary artery disease.

## Figures and Tables

**Table 1:** Clinical characteristics.

		Medical (*n* = 716)	CABG (*n* = 196)	PCA_PCI (*n* = 539)	*p*
Age (years)		58.6 ± 12.4	63.2 ± 10.5	61.8 ± 12.1	0.000
LDL (mg/dL)		96.0 ± 34.1	104.6 ± 41.6	100.0 ± 41.9	0.000

Gender	Male	413 (58%)	152 (78%)	376 (70%)	0.000
	Female	303 (42%)	44 (22%)	163 (30%)	

Diabetic	Yes	213 (30%)	84 (43%)	194 (36%)	0.000
Hypertensive	Yes	512 (72%)	139 (71%)	382 (71%)	0.000
Coronary disease	Yes	191 (27%)	57 (29%)	251 (47%)	0.000

Triglycerides	<150 (mg/dL)	531 (74%)	124 (63%)	336 (62%)	
	150–300 (mg/dL)	158 (22%)	59 (30%)	168 (31%)	
	>300 (mg/dL)	27 (4%)	13 (7%)	35 (76%)	

**Table 2:** Logistic regression model results.

	CABG	PCA_PCI
Age	1.04^*∗∗∗*^ (0.01)	1.03^*∗∗∗*^ (0.01)
Male	3.23^*∗∗∗*^ (0.64)	1.77^*∗∗∗*^ (0.23)
LDL	1.00^*∗∗∗*^ (0.02)	1.00^*∗∗∗*^ (0.01)
Diabetic	1.97^*∗∗∗*^ (0.35)	1.32^*∗∗*^ (0.18)
Hypertensive	0.85 (0.16)	0.75^*∗∗*^ (0.10)
Coronary disease	0.94 (0.18)	2.36^*∗∗∗*^ (0.30)
150–300 mg/dL	1.71^*∗∗*^ (0.32)	1.74^*∗*^ (0.24)
300+ mg/dL	2.48^*∗∗*^ (1.00)	1.55^*∗∗∗*^ (0.50)
Intercept	0.02^*∗∗∗*^ (0.01)	0.02^*∗∗∗*^ (0.12)
*N*	196	539

Total *n* = 1451.

Notes: reference category for the equation is medical management with a triglyceride level less than 150 mg/dL. Standard errors in parenthesis.

^*∗*^ *p* = 0.05, ^*∗∗*^ *p* = 0.01, and ^*∗∗∗*^ *p* = 0.001 (two-tailed tests).

Reported are odds ratios.

**Table 3:** Predicted probabilities.

	Medical		CABG		PCA_PCI	
	M	SD	M	SD	M	SD
<150	0.54	0.15	0.12	0.07	0.34	0.12
150–300	0.41	0.14	0.15	0.08	0.44	0.12
>300	0.38	0.13	0.20	0.07	0.43	0.12
